# Fever as a Predictor of COVID-19 Outcomes in Hospitalized Patients

**DOI:** 10.7759/cureus.54738

**Published:** 2024-02-23

**Authors:** Lucas Pereto Silva, Rebecca Benício Stocco, Marcos Roberto Curcio Pereira, Julia Naomi Koga, Isabela Pontarolo Gomes, João Eduardo Carvalho, Giovana Muniz Beni, Paulo Negreiros, Cristina P Baena, Gustavo Lenci Marques

**Affiliations:** 1 School of Medicine, Pontifícia Universidade Católica do Paraná (PUCPR), Curitiba, BRA; 2 Internal Medicine, Cajuru University Hospital, Curitiba, BRA; 3 Cardiology, Hospital Marcelino Champagnat, Curitiba, BRA; 4 Health Science Postgraduate Program, Pontifícia Universidade Católica do Paraná (PUCPR), Curitiba, BRA; 5 Education, Research and Innovation Center, Hospital Marcelino Champagnat, Curitiba, BRA; 6 Internal Medicine Department, Universidade Federal do Paraná, Curitiba, BRA

**Keywords:** pandemic, curitiba, coronavirus, predictor, hospitalized, body temperature, outcomes, death, covid-19, fever

## Abstract

Introduction: With the advent of the COVID-19 pandemic, numerous questions have arisen regarding the screening, diagnosis, treatment, and prognosis of infected patients. Among these, screening infected patients through body temperature measurement has proven ineffective. However, doubts persist regarding the role of fever as a prognostic factor in the disease.

Objective: To assess the prevalence of fever and its relevance as a marker of mortality in COVID-19.

Methodology: This prospective and longitudinal cohort study was conducted between April 2020 and December 2021 and analyzed 1400 COVID-19 patients systematically admitted to the emergency department of a reference hospital during the period from April 2020 to December 2021, in the city of Curitiba, Brazil. [LG1] The study evaluated [LG2] the presence of fever (body temperature above 37,7ºC) upon admission and/or during hospitalization, patient profiles, and outcomes (in-hospital death, discharge, admission at the intensive care unit, need of mechanical ventilation).

Results: Fever was present in 128 participants (9.1%), with a higher prevalence in males (71%) and obese individuals (42.9%). Among the febrile patients, 39 required intubation (30.4%), with two intubated upon admission (1.5%), 104 were discharged (81.2%), and 24 deceased (18.7%). Fever was not associated with a higher mortality rate.

Conclusion: Fever showed low prevalence, is more common in males and obese individuals, and is not related to worse clinical outcomes.

## Introduction

COVID-19, a severe respiratory illness caused by the SARS-CoV-2 coronavirus, had its first diagnosed case at the end of 2019 in Wuhan, a city in the Hubei province of China [1]. Since then, its spread has escalated to the point of being characterized as an epidemic in China and subsequently crossing borders, becoming a global pandemic.

In terms of clinical manifestations, individuals infected by the agent may develop asymptomatic or symptomatic cases, with the latter generally characterized by fever, cough, myalgia, headache, diarrhea, odynophagia, abnormalities in smell and taste, and dyspnea [2]. In this context, similar to other outbreaks of infectious diseases (such as MERS), temperature measurement as a screening method was widely used by hospitals, pharmacies, markets, and generally in places with a potential for increased population gatherings, posing a higher risk of disease transmission [3].

However, through the conduct of in-depth studies on the screening of febrile patients, Beni et al. found it to be an ineffective method, as fever was often an infrequent or late symptom [4]. Additionally, its measurement was hindered by the use of inadequate or misaligned equipment, the influence of external factors (such as ambient temperature), and the absence of a consensus on the cutoff point for fever identification in different measurement methods, leading to imprecise conclusions. Nevertheless, fever remains a symptom frequently associated with COVID-19.

This raises a new question: what would be the profile of febrile patients in COVID-19? Furthermore, could fever at least be useful as a variable to assess patient prognosis? Hence, this article aims to centrally evaluate, through a prospective and longitudinal cohort study, the prevalence of fever and its relevance as a marker of mortality and morbidity in COVID-19. By exploring the potential to become a predictor of outcomes (in-hospital death, discharge, admission at the intensive care unit, need of mechanical ventilation), the goal is to fill crucial gaps regarding the evolution and prognosis of infected patients, potentially enhancing strategies for the prevention, treatment, and clinical monitoring of COVID-19 and even future viral epidemics.

## Materials and methods

This prospective and longitudinal cohort study was conducted between April 2020 and December 2021 at a tertiary referral center (Hospital Marcelino Champagnat) in Brazil. All patients were over 18 years old; hospital admission with clinical presentation compatible with COVID-19, including dyspnea (SaO2 <92%) and/or cough; immunological or molecular tests confirming COVID-19 infection or other respiratory viruses. The exclusion criteria were a negative test result (RT-PCR or serology) for COVID-19.

All data was prospectively collected through Phillips Tasy medical records. The collected variables were: presence of fever during hospitalization, age, gender, mortality, onset of symptoms chronology, respiratory rate at admission, oxygen saturation in ambient air at admission, need for intense care unit (ICU) during hospitalization, need for orotracheal intubation upon admission and/or during hospitalization, creatinine at admission, body mass index (BMI) ≥ 30 kg/m2, mean diastolic and systolic blood pressure during hospitalization, mean blood glucose during hospitalization, presence of systemic arterial hypertension (SAH), diabetes mellitus (DM), heart failure, previous stroke, chronic obstructive pulmonary disease (COPD), asthma, chronic kidney disease/renal transplant/nephrectomy, anemia, smoking, and the presence of any cardio-metabolic diseases (SAH, DM, cardiac insufficiency, chronic coronary syndrome, myocardial infarct, previous stroke, peripheral arterial disease, dyslipidemia). The primary study outcomes were death, need of ICU, and need of orotracheal intubation during hospitalization.

As a cutoff value for fever, the authors considered a body temperature above 37.8°C (100 °F), measured using infrared thermometers upon admission and daily during hospitalization [5]. Data collection was monitored in real-time by the authors.

The hospital's research ethics committee (Center for Teaching, Research, and Innovation [CEPI]) approved the study protocol on March 20, 2020, and was registered at Plataforma Brasil (CAAE 30188020.7.1001.0020). All patients or their legally authorized representatives signed a consent term before the start of the study.

After gathering the data, it was systematically organized into an Excel spreadsheet for comprehensive analysis. The study employed a convenience sampling method, encompassing all patients who sought emergency care for COVID-19 symptoms during the study period, thus no sample size calculation was conducted. The dataset comprised 27 distinct variables, each representing a unique aspect of the study, such as demographic details, clinical parameters, treatment outcomes, and patient responses. To assess the relationships and differences among these variables, we applied a variety of statistical tests tailored to the nature of each variable. For continuous variables, where we aimed to compare means between two independent groups, the independent samples t-test was utilized. In situations involving categorical variables with two outcomes, Fisher's exact test was employed to determine the significance of associations, especially useful for small sample sizes. For categorical variables with multiple categories, the chi-square test was utilized to explore associations or differences in distribution across groups. This multifaceted statistical approach allowed us to rigorously analyze the data, ensuring that each variable was evaluated using the most appropriate statistical test for its type and distribution.

## Results

In the present study, a total of 1400 patients were included, of whom 869 (62.1%) were male and 531 (37.9%) were female, with an overall mean age of 57 years. Among the participants, 451 were obese (32.2%), 646 were hypertensive (46.1%), 342 had type I or II diabetes (24.4%), and 749 had any cardio-metabolic disease (53.5%). Additionally, 53 patients had COPD (3.7%), 56 were asthmatic (4%), and 140 were smokers (or reformed smokers) (10%).

Regarding the disease history, symptoms began on average 8 days before hospitalization, with an average temperature of 36.5°C at admission and an average oxygen saturation of 92% in ambient air. During hospitalization, 396 patients required ICU beds (28%), with 331 being directly admitted to the ICU (23.6%). Of the total, 394 patients required orotracheal intubation during hospitalization (28.1%), and 57 needed intubation upon admission (4%). As an outcome, 1149 patients were discharged (82.07%), 250 deceased (17.8%), and one participant was transferred to another hospital center (0.7%).

Regarding fever, it was present in 128 participants (9.1%) and was more prevalent in male patients (71%) and obese individuals (42.9%), with statistically significant differences. In the febrile group, 39 required intubation (30.4%), with two being intubated upon admission (1.5%), 104 were discharged (81.2%), and 24 deceased (18.7%), with none transferred to another hospital center. Fever was not associated with a higher mortality rate. All findings are detailed in Table 1.

**Table 1:** General characteristics of the study population SD: standard deviation; SAH: systemic arterial hypertension; DM: diabetes mellitus; COPD: chronic obstructive pulmonary disease; BMI: body mass index; MI: myocardial infarction *Student t-test significance for independent samples, p<0.05 ^#^Fisher's exact test significance, p<0.05 ^§^Chi-square test significance, p<0.05.

Variables	Febrile (n=128)	Afebrile (n=1272)	p value
Age, average±SD	56.69 ± 16.54	57.82 ±16.75	0.468*
Masculine sex, n(%)	91 (71.1%)	778 (61.1%)	0.028#
SAH, n(%)	62 (48.4%)	584 (45.6%)	0.642#
DM, n(%)	34 (26.5%)	308 (24.2%)	0.59#
Cardiac insufficiency, n(%)	3 (2.3%)	68 (5.3%)	0.201#
Previous stroke, n(%)	4 (12.1%)	124 (9%)	0.536#
COPD, n(%)	5 (3.9%)	48 (3.7%)	0.812#
Asthma, n(%)	6 (4.6%)	50 (3.9%)	0.636#
Chronic kidney disease/kidney transplant/nephrectomy, n(%)	7 (5.4%)	48 (3.7%)	0.338#
Usual creatinine/Admission creatinine (mg/dL), average±SD	0.99 ± 0.50	1.03 ± 0.77	0.697*
Anemia, n(%)	3 (2.3%)	35 (2.7%)	1*
Smoking, n(%)			0.241#
Frequent	1 (0.78%)	30 (2.36%)	
Reformed-smoker	15 (11.7%)	94 (7.3%)	
Never smoked	74 (10.6%)	619 (89.3%)	
Days between the beginning of the symptoms and hospitalization (days), average±SD	8 ± 6.22	8.17 ± 4.53	0.696*
Required of ICU during hospitalization, n(%)	60 (9.7%)	68 (8.6%)	0.514
Systolic blood pressure, average±SD	128.67 ± 20.58	127.94 ± 19.99	0.697*
Diastolic blood pressure, average±SD	76.94 ± 12.57	76.86 ± 13.64	0.944*
Admission dextrometry, average±SD	121.88 ± 48.79	150.48 ± 64.26	0.089*
Admission respiratory frequency -(bpm), average±SD	20.55 ± 4.82	20.79 ± 6.45	0.734*
Admission oxygen saturation (SatO_2_) (%), average±SD	92.49 ± 4.03	92.05 ± 6.20	0.273*
Obesity (BMI ≥30kg/m^2^), n(%)	55 (53.4%)	396 (39.4%)	0.008#
Admission orotracheal intubation, n(%)	2 (1.5%)	55 (4.3%)	0.161#
Required orotracheal intubation, n(%)	39 (30.5%)	355 (28%)	0.539#
Cardio-metabolic disease (SAH, DM, cardiac insufficiency, chronic coronary syndrome, myocardial infarct, previous stroke, peripheral arterial disease, dyslipidemia), n(%)	71 (55.4%)	723 (56.8%)	0.779#
Mortality, n(%)	24 (18.75%)	226 (17.8%)	0.809#

## Discussion

In this study, fever was not prevalent and not related to in-hospital death, admission at the ICU, need of mechanical ventilation or discharge. Although, the male gender exhibited a higher frequency of fever than the female gender. Additionally, there are studies [6] that have identified significant differences in clinical outcomes between genders, ranging from a higher rate of ICU admissions and mechanical ventilation use to an increased chance of death in males. The mechanisms justifying this distribution are not well-defined, but it is believed to be related to the expression of genes encoding the angiotensin-converting enzyme 2 receptor and TMPRSS2, responsible for the cellular entry of the SARS-CoV-2 virus [7].

Another extensively discussed aspect in the medical community is the frequency of this symptom across different age groups. In the literature, Remelli et al. identified a low incidence of fever in elderly individuals with COVID-19 [8], attributing it to a possible diminished thermoregulatory response, including sudomotor and vasomotor responses, as well as quantitative and qualitative abnormalities in the production and response to endogenous pyrogens such as IFN-y, IL-4, IL-6, and TNF-α [9]. Apart from advanced age, other comorbidities are also the focus of study in COVID-19. Several known conditions pose a higher risk of developing severe disease, including evidence that the presence of asthma, COPD [10], chronic kidney disease (as well as patients on dialysis) [11], type 1 and 2 diabetes [12,13], cardiovascular diseases (such as heart failure, coronary artery disease, or cardiomyopathies) [14], obesity [15] (BMI > 30 kg/m2 or > 95th percentile in children), and current or former smoking [16] have a stronger association with poor prognosis in patients infected with the SARS-CoV-2 virus. Additionally, a cross-sectional study conducted in the USA in March 2021 [16], based on data from 540,667 adults hospitalized with COVID-19 in 592 hospitals, concluded that the presence of multiple comorbidities is linked to severe disease, and obesity, diabetes with complications, and disorders related to anxiety and fear had the strongest association with death.

In the present study, a higher frequency of fever was identified in obese patients. Nevertheless, there is no prior study associating the frequency of fever with the presence of comorbidities to assess which patients are at risk and how this might influence the mortality rate.

Among the highlighted comorbidities is obesity (BMI > 30 kg/m²), which has been a subject of study in various research endeavors related to COVID-19. Frequently, obesity is associated with other comorbidities such as hypertension, DM, and cardiovascular diseases, resulting in the intensification of pro-inflammatory events and alterations in metabolism and the endocrine system [17-20].

After data analysis, more than half (53%) of the patients who developed fever during hospitalization were obese. In line with a study conducted in the state of Alagoas, Brazil [21], which assessed the disease profile in 138 cases of COVID-19 in obese patients and identified fever in more than half of the study group (56.5%). A similar finding was reported by Alqahtani et al. [22], who described fever as one of the most prevalent symptoms in overweight patients (BMI 25-29.9 kg/m²). Moreover, various studies have already identified obesity as an isolated risk factor for a higher rate of hospitalization, longer hospitalization time, the need for mechanical ventilation, and a fatal outcome [23-26].

Regarding outcomes, the present study did not show a significant relationship between the presence of fever and the need for intubation, ICU admission, or death. However, it is believed that other factors may be associated with this outcome, not just the presence of fever. Even though there is not a wide range of studies correlating the presence of comorbidities and pre-existing conditions with the in-hospital outcome of patients, findings of worse outcomes in patients are frequent with type 2 diabetes, hypertension, dyslipidemia, COPD, chronic kidney disease, the elderly, males, and especially obese individuals [6,7,27-29].

Since some of these populations present fever more frequently, perhaps this is not an isolated factor for a worse prognosis but rather its association with other illnesses. The importance of conducting future studies to better understand the disease profile based on the presence of the most common comorbidities in COVID-19 patients is emphasized.

The limitations of the research include patient omission or lack of knowledge about their comorbidities or pre-existing conditions, the possibility of incomplete or improperly filled out medical records by healthcare professionals, incorrect vital sign data measurement, or the use of uncalibrated/inadequate equipment, lack of diagnostic tests to confirm reported comorbidities, and infection by strains that do not cause fever.

## Conclusions

The findings of our investigation suggest that fever, while clinically observable, should not be relied upon as a singular screening tool for SARS-CoV-2 infection. Despite its occurrence in a minority of the patient population, its predictive value for hospitalization outcomes was statistically insignificant. Moreover, fever's prevalence did not align with higher mortality rates, indicating its limited utility in prognostic stratification. These insights underscore the necessity for a multifaceted approach to screening and risk assessment in the clinical management of this disease, with an emphasis on a broader range of symptoms and biomarkers for a more effective triage and therapeutic intervention strategy. Further exploration into the complex symptomatology and patient demographics is imperative for the development of refined diagnostic criteria and treatment protocols.
